# Intact revision rotator cuff repair stabilizes muscle atrophy and fatty infiltration after minimum follow up of two years

**DOI:** 10.1186/s12891-023-06616-2

**Published:** 2023-06-23

**Authors:** Lucca Lacheta, Sebastian Siebenlist, Bastian Scheiderer, Knut Beitzel, Klaus Woertler, Andreas B. Imhoff, Stefan Buchmann, Lukas Willinger

**Affiliations:** 1 Department of Orthopaedic Sports Medicine, Technical University of Munich, Ismaninger Str. 22, 81675 Munich, Germany; 2 ATOS Orthoparc Klinik, Cologne, Germany; 3 Musculoskeletal Radiology Section, TUM School of Medicine, Technical University of Munich, Ismaninger Str. 22, 81675 Munich, Germany; 4 Orthopaedisches Fachzentrum (OFZ) Weilheim/Garmisch/Starnberg/Penzberg, Weilheim, Germany

**Keywords:** Rotator cuff, Supraspinatus tendon, Fatty infiltration, Rotator cuff atrophy, Revision rotator cuff repair

## Abstract

**Background:**

The extent of fatty infiltration and rotator cuff (RC) atrophy is crucial for the clinical results after rotator cuff repair (RCR). The purpose of this study was to evaluate changes in fatty infiltration and RC atrophy after revision RCR and to correlate them with functional outcome parameters.

**Methods:**

Patients who underwent arthroscopic revision RCR for symptomatic recurrent full-thickness tear of the supraspinatus tendon between 2008 and 2014 and were retrospectively reviewed with a minimum follow up of 2 years. Magnetic resonance imaging (MRI) was performed pre- and postoperatively to assess 1) tendon integrity after revision RCR according to Sugaya classification, (2) RC atrophy according to Thomazeau classification, and (3) fatty infiltration according to Fuchs MRI classification. Constant score (CS) and the American Shoulder and Elbow Surgeon (ASES) score were used to correlate functional outcome, tendon integrity, and muscle degeneration.

**Results:**

19 patients (17 males and 2 females) with a mean age of 57.5 years (range, 34 to 72) were included into the study at a mean follow-up of 50.3 months (range, 24 – 101). At final evaluation, 9 patients (47%) presented with intact RCR and 10 patients (53%) suffered a re-tear after revision repair. No progress of fatty infiltration was observed postoperatively in the group with intact RC, atrophy progressed in only 1 out of 9 patient (11%). Fatty infiltration progressed in 5/10 patients (50%) and RC atrophy increased in 2/10 patients (20%) within the re-tear group. CS (42.7 ± 17.7 preop, 65.2 ± 20.1 postop) and ASES (47.7 ± 17.2 preop, 75.4 ± 23.7 postop) improved significantly from pre- to postoperatively (*p* < 0.001). A positive correlation between fatty infiltration and RC integrity was detected (r = 0.77, *p* < 0.01). No correlation between clinical outcome and tendon integrity or RC atrophy was observed.

**Conclusion:**

Arthroscopic revision RCR leads to reliable functional outcomes even in case of a recurrent RC retear. An intact RCR maintains the preoperative state of fatty infiltration and muscle atrophy but does not lead to muscle regeneration.

**Level of evidence:**

Level IV; Therapeutic study.

## Background

Full-thickness rotator cuff (RC) tears lead to tendon and muscle degeneration over time. The degeneration process includes retraction of the tendon as well as atrophy and fatty infiltration of the muscles [1, 2]. The pathophysiology of these progressive changes is still unclear and subject of recent research [3–5].

The extent of fatty infiltration and RC atrophy is crucial for the clinical results after rotator cuff reconstruction (RCR). Advanced degenerative changes are associated with poor clinical outcome and higher failure rate after RCR [1, 6–8]. The course of initiated degenerative changes after RCR is still debated. While some author reported stabilization of degeneration after successful RCR, others postulate an irreversible process with worsening of fatty infiltration and RC atrophy over time [1, 6, 7, 9].

Recently, Deniz et al. demonstrated stabilization of RC atrophy and fatty infiltration after primary RCR when rotator cuff integrity was maintained. In case of re-tear the authors showed that fatty infiltration and atrophy continued to deteriorate [10]. After revision surgery, tendon integrity appears to be a problem despite a good clinical outcome. In short-term follow-up, ultrasound and MRI controlled examinations indicated only a 48% to 60% healing rate [11–13].

There is no information whether revision RCR prevents further muscle atrophy and fatty infiltration after failed primary repair. Therefore, the purpose of this study was to evaluate changes in fatty infiltration and muscle atrophy after revision RCR and to correlate them with functional outcome parameters. It was hypothesized that progression of fatty infiltration and RC atrophy can be preserved after successful revision and that tissue quality and repair integrity positively correlates with clinical outcome.

## Methods

### Study population

A retrospective review with prospective clinical and radiological evaluation with a minimum follow-up of 24 months was performed. Patients who [1] underwent arthroscopic revision RCR of [2] the supraspinatus tendon between 2008 and 2014 were included. Revision surgery was indicated due to [3] residual pain, loss of strength or function of the affected arm and [4] full-thickness supraspinatus re-rupture on MRI. These subjects were identified from a previous series of revision repair patients with shoulder magnetic resonance imaging obtained at least 24 months postoperatively [13]. Patients with glenohumeral osteoarthritis exceeding Samilson and Prieto grade I were excluded from analysis. Demographic and surgical data were documented from medical records, operation report and follow up notes. The present study was approved by the Institutional Review Board (No.: 128/16 S) and conducted according to the Declaration of Helsinki. All patients gave their written informed consent.

### Clinical outcome assessments

All patients underwent clinical assessments prior to arthroscopic revision RCR and at final follow-up evaluation. Constant shoulder score (CS) and the American shoulder and elbow score (ASES) were used to measure subjective outcome. Pain was scaled with the visual analog pain score (VAS). Furthermore, patients were asked if they were satisfied with the clinical outcome after revision repair (very satisfied, satisfied, not satisfied).

### Radiological evaluation

All patients underwent standardized radiographs (true a.p., y-view, axial) before surgery to rule out glenohumeral osteoarthritis (OA) exceeding state I according to Samilson and Prieto [14]. Magnetic resonance imaging (MRI) of the affected shoulder was performed prior to surgery and at the final-up evaluation at least 24 months after arthroscopic revision RCR. Postoperative MRI was performed using a 3-Tesla scanner and a dedicated 8-channel shoulder coil (Ingenia, Philips, Best, The Netherlands). The following pulse sequences were acquired with a section thickness of 3 mm: parasagittal T1- and fat-suppressed intermediate weighted turbo spin echo (TSE) sequences, parasagittal T2-weighted and fat-suppressed intermediate weighted TSE sequences, and a transverse intermediate weighted TSE sequence with fat suppression. Both MRI examinations were evaluated by two independent orthopaedic senior residents (L.W., L.L.) and one radiologic consultant (K.W.) specialized in musculoskeletal radiology listed as authors.

First the integrity of the supraspinatus tendon was assessed by using the classification of Sugaya et al. [15]: Type I, intact tendon repair without inhomogeneous signal; type II, intact tendon repair with high signal in partial area suggesting degeneration; type III, thinned tendon without discontinuity classified as partial retear; type IV, small RC re-tear and type V major discontinuity. Type I to III were rated as an integrated supraspinatus tendon, type IV and V as a re-tear.

Fatty infiltration was classified according to Fuchs et al. [16]: Grade 0, no fatty infiltration; Grade 1, some fatty streaks in the supraspinatus muscle; Grade 2, less than 50% of fat compared to muscle; Grade 3, equal amounts of fat and muscle; Grade 4, more fat, less muscle.

Atrophy of the supraspinatus muscle was classified and measured according to the Thomazeau classification [9]. The estimated anatomical surface of the supraspinatus muscle (fossa supraspinatus) and the actual surface of the supraspinatus muscle were measured. The occupation ratio was calculated. Due to the occupation ratio, muscle atrophy was rated: Stage 1, normal atrophy occupation ratio (1.0–0.6); Stage 2, moderate atrophy occupation ratio (0.6–0.4); Stage 3, severe atrophy occupation ratio (< 0.4).

All measurements were performed on oblique sagittal T1-weigthed MR images at the most medial sagittal section that shows a Y-shaped configuration of the scapula. The time from preoperative MRI to surgery was on average 50 ± 37 days (range, 2–127).

### Surgical technique and postoperative rehabilitation

All patients were operated or directly supervised by the senior surgeon (A.B.I.). Surgery was performed in general anesthesia without interscalene nerve block in beach chair position. Preparation and draping using sterile technique followed diagnostic arthroscopy. If not already done in prior surgery, the long head of the biceps tendon underwent either tenodesis or tenotomy. Tear size and configuration of the supraspinatus tendon were evaluated. Subacromial release of adhesions and bursectomy was performed and the supraspinatus tear was debrided. An extensive release of the supraspinatus tendon was done intraarticular and subacromial until tension-free mobility/traction of the tendon was achieved. To enhance healing decortication of the greater tuberosity was performed by using a shaver until bleeding of the debrided bone was visible. Depending on tear size, four patients were operated with single-row and 15 with double-row (crossing suture-bridge) configuration with double loaded anchors (Titan-Corkscrew or Bio-Corkscrew 5.5 mm). In all patients, a complete reduction of the supraspinatus tear to the anatomical footprint was achieved.

For postoperative management, all patients had the affected arm secured in an arm orthesis with 30° of abduction for 4–6 weeks. Pain medication was utilized as required. Physical therapy started at postoperative day one with passive flexion and abduction and free range of motion (Abduction was limited not to be less than 30°). Active-assisted exercises were allowed in postoperative week 7 and active movements after 9 weeks. Physical therapy with a professional therapist took place 2 to 3 times per week for at least 3 months after surgery.

### Statistical analysis

Statistical analysis was performed with SPSS version 22.0 (SPSS, Chicago, IL). Kolmogorov–Smirnov test was used to assess data for normal distribution. In this data set, continuous variables were normally distributed, except postoperative ASES, and stated as mean and standard deviation. Categorical variables were stated as percentages and frequency distribution. Students t-test was used to calculate differences of continuous variables (pre- and postoperative scores). In order to find a correlation between tendon integrity and clinical outcome scores Spearman correlation test was used. Fisher’s exact test was utilized to study differences in pre- and postoperative categorical variables (RC atrophy and fatty infiltration). ICC was calculated for inter-rater reliability. Statistical significance was set at a *p* value of < 0.05.

## Results

### Clinical outcome

19 patients (17 males and 2 females), with a mean age of 57.5 years (range, 34 to 72) were surveyed at a mean follow-up of 50.3 months (range, 24 – 101, see also Table 1). At final follow up the mean ASES score improved from 47.73 ± 17.2 preoperatively to 75.4 ± 23.7 postoperatively (*p* < 0.001), the mean CS from 42.7 ± 17.7 preoperatively to 65.2 ± 20.1 postoperatively (*p* < 0.001) and the mean VAS significantly from 6.0 ± 1.8 preoperatively to 1.6 ± 2.1 postoperatively (*p* < 0.001). Improvements exceeded MCID of all scores. At final follow up 14 patients (74%) were very satisfied, 3 patients (16%) satisfied and 2 patients (10%) not satisfied. There was no preoperative difference in CS (39.0 ± 28.3 vs. 44.9 ± 10.4, *p* > 0.05) but in ASES (29.7 ± 10.9 vs. 58.0 ± 9.7, *p* < 0.01) between the intact and re-tear group. Tendon retraction according to Patte and preoperative tendon length was similar between the two groups (*p* > 0.05). However, re-tear classification according to Sugaya showed larger preoperative re-tears in patients who were in the re-tear group compared to the successful repair group (*p* < 0.05).

**Table 1:** Preoperative comparison of demographic factors, clinical scores and rotator cuff status on MRI between the patients with healed or failed rotator cuff revision surgery

		Healed Rotator Cuff Revision	Failed Rotator Cuff Revision	*p*-value
Age (years)		53.7 ± 10.6	61.0 ± 10.1	n.s
BMI (kg/m^2^)		26.8 ± 4.1	26.7 ± 4.1	n.s
Preoperative ASES		29.7 ± 10.9	58.0 ± 9.7	< 0.01
Preoperative Constant		39.0 ± 28.3	44.9 ± 10.4	n.s
Sex	Male	7	10	n.s
	Female	2	0	
Fatty infiltration (Fuchs)	Grade 0	3	0	n.s
	Grade 1	5	8	
	Grade 2	1	2	
RC atrophy (Thomazeau)	Grade 0	5	1	n.s
	Grade 1	2	4	
	Grade 2	2	5	
Tendon retraction (Patte)	Grade 0	3	3	n.s
	Grade 1	4	4	
	Grade 2	1	2	

Postoperative abduction was 155 ± 34° in intact RC and 145 ± 39° in patients with a retear. Patients with an intact supraspinatus tendon exhibited abduction strength of 61.6 ± 34.3N, whereas patients with re-tear showed abduction strength of 42.2 ± 28.0N (*p* > 0.05).

### Radiological outcome

Preoperative re-tears before revision were classified as follows: 9 patients had a type V (major discontinuity) and 10 patients a type IV (small full-thickness RC re-tear) re-tear of the supraspinatus tendon according to the Sugaya classification. Preoperative tendon retraction according to Patte was: 7 patients grade I, 9 patients grade II and 3 patients grade III. 3 patients (16%) presented with no fatty infiltration, 13 patients (68%) with grade 1 and 3 patients (16%) with grade 2 prior to revision surgery.

At final follow-up, 9 patients (47%) had an intact supraspinatus tendon (4 patients Sugaya type II; 5 patients Sugaya type III) and 10 patients (53%) presented with a re-ruptured supraspinatus tendon (3 patients Sugaya type IV; 7 patients Sugaya type V). Re-tear location was at the enthesis in 6 patients, at the musculotendinous junction in 3 patients and intratendinous in 1 patient.

**Fig. 1:**
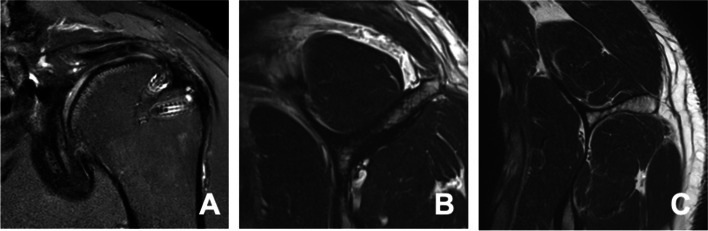
**A** Postoperative coronal MRI showing intact supraspinatus tendon fixed in double-row technique. **B** Preoperative parasagittal MRI of supraspinatus muscle showing good muscle quality slight atrophy due to retraction. **C** Postoperative parasagittal MRI of supraspinatus muscle showing no progression of muscle atrophy or fatty infiltration

**Fig. 2:**
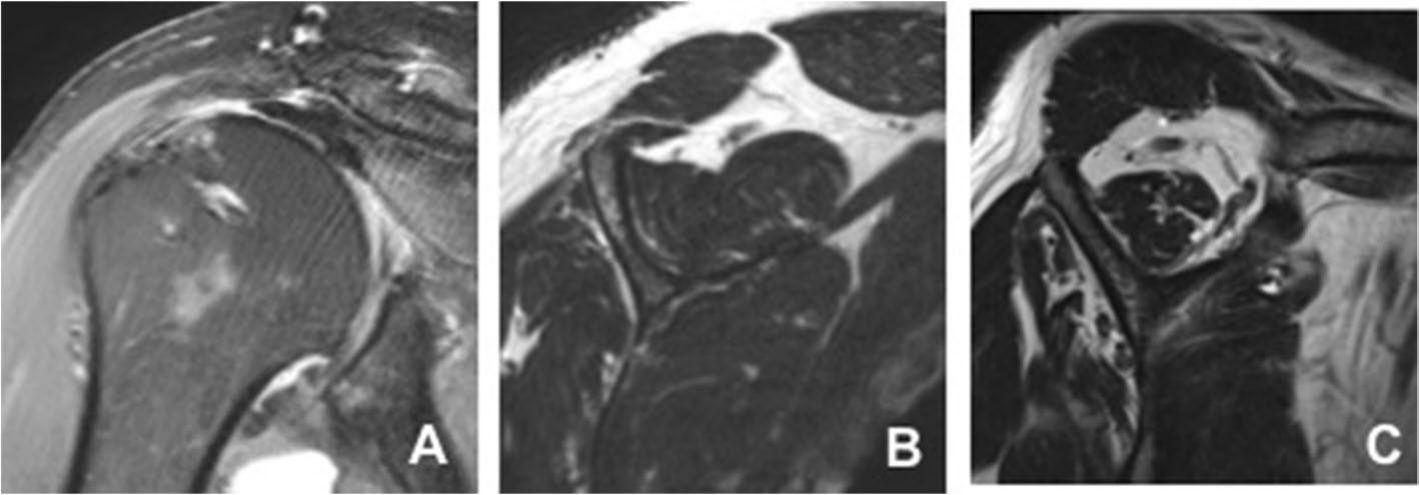
**A** Postoperative coronal MRI showing a supraspintatus tendon re-tear at final follow up (Sugaya Type IV). **B** Preoperative parasagittal MRI of supraspinatus muscle showing slight atrophy. **C** Postoperative parasagittal MRI of supraspinatus muscle showing progression of atrophy and fatty infiltration due to RC retear

The group with an intact supraspinatus tendon postoperatively presented with no difference in fatty infiltration compared to preoperative status and a decrease of atrophy in one patient (1/9 patients, 11%, Fig. 1). In contrast, 5 out of 10 patients (50%) with recurrent RC tears showed progression in fatty infiltration (Fig. 2). RC atrophy showed an advance in 2/10 patients (20%) at time of follow up in the re-tear group. A decrease of fatty infiltration was not recognized in any patient. There was no statistical difference observed preoperatively to postoperatively with regard to these measurements. Table 2 and Table 3 summarize the changes over time in fatty infiltration and atrophy of the supraspinatus muscle observed in the evaluated patients, respectively. The severity of postoperative fatty infiltration or muscle atrophy were not associated with better or worse clinical outcome scores (*p* > 0.05). The Intraclass correlation coefficient (ICC) was 0.89 (CI 95%, 0.70–0.96) for inter-rater agreement for the classification of Sugaya.

**Table 2:** Changes of fatty infiltration according to Fuchs classification in evaluated patients

Intact RC				Re-ruptured RC			
grade	pre-operative	post-operative	*p*-value	grade	pre-operative	post-operative	*p*-value
0	3	3	n.s	0	n	n	n.s
1	5	5		1	8	4	
2	1	1		2	2	1	
3	n	n		3	n	2	
4	n	n		4	n	3	

^*^n none

*n.s* not significant

**Table 3:** Changes in muscle atrophy according to Thomazeau classification in evaluated patients

Intact RC				Re-ruptured RC			
grade	pre-operative	post-operative	*p*-value	grade	pre-operative	post-operative	*p*-value
0	n	n	n.s	0	n	n	n.s
1	5	6		1	1	1	
2	2	1		2	4	2	
3	2	2		3	5	7	

^*^n none

*n.s* not significant

### Correlations

When comparing functional results in patients with intact rotator cuff and re-tear, both groups improved in clinical outcome parameters pre- to postoperatively (*p* < 0.001). No differences at final follow-up for CS and ASES were observed between the groups. Improvements in CS and ASES score were similar in both groups (CS 66.3 ± 24.5 vs. 64.4 ± 17.9, ASES 74.7 ± 22.3 vs. 76.0 ± 25.9; n.s.). Preoperative tendon retraction, RC atrophy or fatty infiltration were not correlated with tendon healing in this cohort. No correlation between RC integrity and clinical outcome as well as supraspinatus atrophy was detected. A strong correlation (Correlation coefficient R = 0.77, *p* = 0.01) between fatty infiltration and cuff integrity was observed.

## Discussion

The study results demonstrate that the status of supraspinatus muscle atrophy and fatty infiltration can be preserved after revision RCR provided that the repair remains intact. However, a regress of fatty infiltration could not be observed. Good to excellent clinical results and high patient satisfaction were observed after arthroscopic revision RCR regardless of the RC integrity.

There are several MRI studies focusing on the tear pattern and outcome after RCR failure [17–19]. Trantalis et al. [17] reported on a small series of five patients with RC re-tears following primary double-row rotator cuff repair and found re-tearing medial to the anchor reconstruction at the musculotendinous junction. In agreement with these findings, Hayashida et al.[18] described a prevalence of medial row failure in 7 of 13 (54%) re-tears within a group of 47 (15%) cases. While the rotator cuff construct seems not to be the limiting factor for long-term healing, the focus of interest switched to degenerative status of the muscle and tendon. A rising number of rotator cuff repairs in elderly with decreased tissue quality could be observed [19, 20]. Djurasovic et al. reported on 24 patients (30%) with poor tendon quality in revision rotator cuff repair (rated subjectively at time of surgery), postulating that the muscle and tendon undergo an intrinsic degeneration [19]. Et al. showed that the state of fatty infiltration (especially in the infraspinatus muscle) could be a reference for successful cuff healing after primary RCR [21].

With regard to outcome after revision RC repairs only few studies reported on clinical outcomes; whereof seven used an arthroscopic approach [11, 12, 19, 22–28]. The functional results and patient satisfaction improved in all series in similar amount to the findings in this study. The prevalence of RCR failure in ultrasound studies ranged from 0 – 62% [11, 12, 26, 29]. The results from the present study after arthroscopic revision repair evaluated by MRI showed a re-tear rate of 53% which is slightly above the average rate reported in the literature. This could be due to the long follow up period since Shamsudin et al. reported an increased number of re-tears over time with a prevalence of 28% after 6 months and 40% two years after surgery [12].

Previous studies reported on pre- and postoperative changes in fatty infiltration and RC atrophy after primary repair with inconsistent findings [1, 6, 9, 30].

First, Goutallier et al. as well as Thomazeau et al. reported promising results with a reduction of fatty infiltration in the supraspinatus muscle in up to 10% of patients with an intact RCR [1, 6, 9]. Yamaguchi et al. showed an reduction of fatty infiltration in 25% and of RC atrophy in 50% of patients with intact primary RCR [30]. The authors underlined the importance of a sufficient follow-up period to detect these changes. These findings were supported by animal studies: Coleman et al. demonstrated a decrease of fatty infiltration in the early follow-up period after RCR [31]. Despite the promising results described in previous studies, we did not find a reduction of fatty infiltration and a decrease in muscle atrophy in only one patient. Provided that the rotator cuff remains intact after revision repair, the state of degeneration could, however, be preserved.

These findings are in agreement with studies from Gerber [32], Gupta [33] and Liu [34] who could not find a reduction in muscle degeneration in animal studies. These studies showed that fatty infiltration starts 6 months after the occurrence of RC tear but could be stabilized when tendon integrity was restored, [32–34]. Lee et al. showed an increase of fatty infiltration in all patients from pre- to postoperative in their short-term computed tomography follow-up of 12.7 months [35]. These findings were supported by Bartl et al. in patients with massive rotator cuff tears showing a progress of fatty infiltration in all patients [36]. Gladstone et al. demonstrated a progression of fatty infiltration in all their patients, but the degree was significantly higher in patients with RC re-tear at time of follow-up [7]. We believe that fatty infiltration and RC atrophy is an almost irreversible process where, in the best possible case, the status of fatty infiltration and RC atrophy can be preserved but not reversed.

Postoperative tendon integrity seems to be the best predictor for progression of muscle degeneration after primary as well as revision RCR. Interestingly, no difference in clinical outcome was observed in both groups (intact vs. re-tears) that could be explained by the possible restoration of the force couple, which preserves good clinical results and patient satisfactory as reported before by Jost et al. and Paxton et al. [37, 38]. Furthermore, the good clinical outcome in the re-tear group might be explained by decreased tear size or intensive postoperative rehabilitation.

The present study has several strengths, but also limitations. The follow-up period was at least 24 months; and with a mean of 50.3 months the follow-up was sufficient to detect changes in RC degeneration. Both, the pre- and postoperative MRI was evaluated and rated by both the orthopedic surgeon and the musculoskeletal radiologist. Additionally, all arthroscopic revision RCR were performed or supervised by one senior surgeon (*blinded for review*) at one single institution.

However, the study has some weaknesses including a heterogeneous cohort of evaluated patients. The small number of individuals was given by the fact that revision RCR is recommended and performed only in selected patients with high functional demands and good tissue quality. This leads to a selection bias due to preoperative subject analysis for reparability of the rotator cuff, tendon and muscle quality. Results could be affected by confounders which have not been analysed due to the small number of patients. Further standardized prospective investigations with larger population cohort are needed to determine higher grade of scientific evidence.

## Conclusion

Arthroscopic revision rotator cuff repair resulted in good to excellent functional outcomes even in case of recurrent RC retear. An intact RCR maintains the preoperative state of fatty infiltration and RC atrophy but does not result in tissue regeneration. Rotator cuff integrity showed a moderate correlation with fatty infiltration.

## Data Availability

The datasets used and/or analysed during the current study available from the corresponding author on reasonable request.
